# Nucleolar stress enhances lytic reactivation of the Kaposi’s sarcoma-associated herpesvirus

**DOI:** 10.18632/oncotarget.24497

**Published:** 2018-02-15

**Authors:** Anastasia Gelgor, Chen Gam ze Letova, Yana Yegorov, Inna Kalt, Ronit Sarid

**Affiliations:** ^1^ The Mina and Everard Goodman Faculty of Life Sciences and Advanced Materials and Nanotechnology Institute, Bar Ilan University, Ramat-Gan, Israel

**Keywords:** Kaposi’s sarcoma-associated herpesvirus, KSHV, lytic reactivation, nucleolar stress, p53

## Abstract

Kaposi’s sarcoma-associated herpesvirus (KSHV) is a human tumorigenic virus exhibiting two forms of infection, latent and lytic. Latent infection is abortive and allows the virus to establish lifelong infection, while lytic infection is productive, and is needed for virus dissemination within the host and between hosts. Latent infection may reactivate and switch towards the lytic cycle. This switch is a critical step in the maintenance of long-term infection and for the development of KSHV-related neoplasms. In this study, we examined the effect of nucleolar stress, manifested by failure in ribosome biogenesis or function and often coupled with p53 activation, on lytic reactivation of KSHV. To this end, we induced nucleolar stress by treatment with Actinomycin D, CX-5461 or BMH-21. Treatment with these compounds alone did not induce the lytic cycle. However, enhancement of the lytic cycle by these compounds was evident when combined with expression of the viral protein K-Rta. Further experiments employing combined treatments with Nutlin-3, knock-down of p53 and isogenic p53+/+ and p53-/- cells indicated that the enhancement of lytic reactivation by nucleolar stress does not depend on p53. Thus, our study identifies nucleolar stress as a novel regulator of KSHV infection, which synergizes with K-Rta expression to increase lytic reactivation. This suggests that certain therapeutic interventions, which induce nucleolar stress, may affect the outcome of KSHV infection.

## INTRODUCTION

The human body plays host to multiple microbial cells, including fungi, bacteria and archaea. These microorganisms, together with their accompanying viruses and with viruses that infect human cells, form our microbiome, which shows interpersonal variation and dynamism. Generally, there is a delicate balance between the host and its microbiome; however, disruptions of this balance, due to changes in the host or microbiome, may lead to disease development [1, 2].

Herpesviruses are common components of the human microbiome [3]. Two herpesviruses are causally associated with cancer in humans: Epstein Barr virus (EBV, also termed human herpesvirus 4 (HHV-4)) and Kaposi’s sarcoma-associated herpesvirus (KSHV, also referred to as human herpesvirus 8 (HHV-8)). KSHV is linked to Kaposi’s sarcoma (KS), primary effusion lymphoma (PEL), the plasmablastic variant of multicentric Castleman’s disease (MCD), and a recently described clinical entity termed KSHV-inflammatory cytokine syndrome [4–7]. In common with all other herpesviruses, following primary infection, KSHV becomes a persistent part of the microbiome and establishes long-term latent infection which can reactivate towards the lytic cycle [8–10]. Physiological factors involved in KSHV reactivation are only partially known [10], and reported inducers include hypoxia [11–13], oxidative stress [14], activation of cellular kinases [15–18], epigenetic alterations [19–22], inflammation [23, 24], and viral co-infection [25, 26]. Experimentally, KSHV reactivation can be chemically induced with inhibitors of histone deacetylases (HDACs) such as n-Butyrate [27, 28], inhibitors of DNA methylation [29] and kinase agonists [27]. Nevertheless, initiation of the lytic cycle proceeds mainly through the triggering of the expression of the viral replication and transcription activator protein (K-Rta), which in-turn, positively autoregulates itself and activates an expression cascade of lytic genes which is coupled with extensive viral DNA replication, and production of infectious progeny virions [30–32].

The KSHV transition from latent to lytic infection is a critical step for virus dissemination within the host and between hosts, for the maintenance of lifelong infection, and for the progression and pathogenesis of KSHV-related neoplasms [10, 33]. Accordingly, increased loads of KSHV in the PBMCs, coupled with compromised immunological surveillance, is strongly associated with the development of KSHV-related diseases [34–37]. Furthermore, although KSHV is present mainly in its latent form in KS lesions, small subpopulations of cells undergo lytic replication and are thought to support the initiation and progression of KS [38–41]. Similarly, PEL is also considered a disease of latency, though small fractions of cells undergo lytic infection and produce paracrine factors [40, 42–44].

Previous studies employing latently KSHV-infected cells have shown that inhibition of MDM2 by Nutlin-3, as well as genetic knockdown of MDM2 [45], result in activation of the tumor suppressor p53 pathway and stabilization of K-Rta leading to cell cycle arrest, massive cell death and disruption of viral latency. Based on these observations, we hypothesized that nucleolar stress, described as failures in ribosome biogenesis or function, which are often coupled with inhibition of MDM2 and p53 activation, might promote lytic reactivation of KSHV. We report here that while induction of nucleolar stress by a series of compounds did not stimulate lytic reactivation of KSHV, these compounds enhanced lytic cycle induction of KSHV when combined with expression of the K-Rta protein. Further experiments employing combined treatments with Nutlin-3, knock-down of p53 by siRNA and isogenic p53+/+ and p53-/- cells indicated that enhancement of lytic reactivation by nucleolar stress does not depend on p53.

## RESULTS

### Nucleolar stress produced by Actinomycin D enhances lytic reactivation of KSHV

To examine the effect of nucleolar stress on lytic reactivation of KSHV, we used human epithelial iSLK cells (expressing wild-type p53) that were engineered to express a doxycycline (Dox)-inducible K-Rta protein, and were previously employed to study lytic induction of latent KSHV infection [46, 47]. These cells were infected with a recombinant dual fluorescent KSHV (BAC16-mCherry-ORF45) encoding green fluorescent protein (GFP) under the control of the constitutive cellular promoter EF1α, and the lytic tegument protein ORF45 fused to monomeric cherry fluorescent protein (mCherry) [48]. In this experimental setting, expression of GFP can be used to identify infected cells, while expression of mCherry identifies cells undergoing lytic infection. This reporter system is similar to that used in the previously reported KSHV.219 recombinant virus, in which the red fluorescent protein (RFP) gene is expressed from the lytic PAN RNA promoter, used as a marker for lytic induction [49].

**Figure 1:**
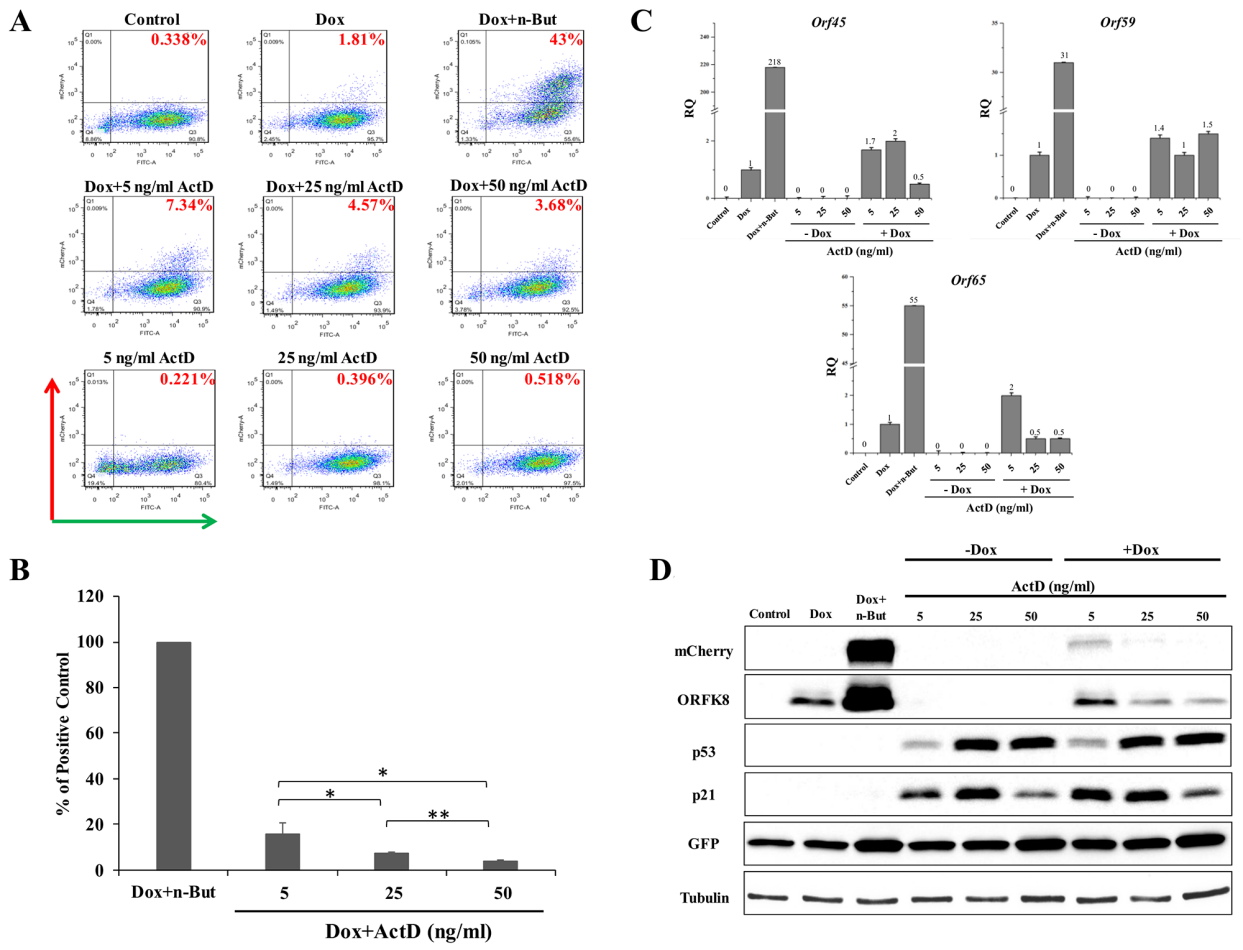
Enhancement of lytic induction of KSHV by combined treatment with Actinomycin D (Act D) and doxycycline (Dox) in iSLK cells **(A)** BAC16-mCherry-ORF45-infected iSLK cells were left untreated (control) or treated with Act D (5, 25 or 50 ng/ml) alone or in combination with 1μg/ml Dox for 48 hours. Treatment with Dox alone (Dox) and combined treatment with Dox and 1 mM n-Butyrate (Dox+n-But) were used as references. Lytic virus reactivation was assayed by FACS analysis of GFP and mCherry expressing cells (Representative experiment). **(B)** Average of three independent experiments relative to treatment with Dox+n-But ±s.d. The percentage of mCherry-positive cells that were detected following Dox treatment was first subtracted from the percentage that was detected following each treatment. Results are presented relative to the positive control, the percentage of mCherry-positive cells that were detected following incubation with Dox+n-But (normalized to 100%). ^*^p<0.05, ^**^p<0.01. **(C)** RT-qPCR for the viral lytic transcripts *orf45*, *orf59* and *orf65* representing immediate-early, early and late genes, respectively. mRNA abundance after induction is shown relative to Dox treatment, which was defined as 1 after normalization. **(D)** Cell extracts were subjected to western blot analysis with antibodies to mCherry, ORFK8, p53, and its target p21, GFP and Tubulin. RT-qPCR and western blot results represent one experiment representative of four, providing similar results.

First, we wished to determine the impact of Actinomycin D (Act D) on lytic reactivation of KSHV. Act D is a well-characterized inhibitor of all three eukaryotic RNA polymerases. Yet, RNA polymerase I transcription is by far the most sensitive to Act D (inhibited at a dose of 5-50 ng/ml), followed by RNA polymerase II (500 ng/ml) and RNA polymerase III (∼5 μg/ml). At low concentrations, Act D intercalates preferentially within GC-rich rDNA to selectively block RNA polymerase I transcription elongation. Thus, treatment with this compound at low doses inhibits RNA polymerase I transcription but does not reduce the activities of other RNA polymerases [50, 51]. Cells were treated with 5, 25 and 50 ng/ml Act D alone, or together with Dox (to induce K-Rta), for 48 hours, and the percentage of GFP and mCherry-positive cells was determined by FACS analysis. As shown in Figure 1A and in line with previous results, under standard growth conditions nearly all cells expressed GFP and almost no mCherry-expressing cells were observed, indicating that most cells harbor latent infection. This feature is common with cultures of PEL cells that are predominantly latently infected under standard growth conditions [42, 43]. A small increase in the percentage of cells expressing mCherry was evident after induction of K-Rta expression by Dox treatment. In line with previous reports, the effect of Dox was substantially augmented when combined with the histone deacetylase (HDAC) inhibitor, n-Butyrate [46, 48]. Treatment with Act D alone failed to increase the fraction of cells expressing mCherry. Yet, Act D slightly enhanced the effect of Dox, and higher percentages of mCherry-positive cells were detected 48 hours after combined treatment. Averages of repeated independent FACS experiments revealed that treatment with 5 ng Act D was significantly more effective than the other doses tested (Figure 1B). To further confirm lytic induction of KSHV, we examined by reverse-transcription (RT)-quantitative PCR (RT-qPCR), expression of the viral transcripts *orf45*, *orf59* and *orf65* representing immediate-early, early and late viral lytic genes, respectively (Figure 1C). A small increase in the expression of all lytic transcripts was evident in cells that were treated with Act D and Dox, whereas treatment with Act D alone did not result in increased expression of these lytic transcripts. Similarly, increased expression of mCherry-ORF45 and ORFK8 proteins was detected by western blot analysis following combined treatment with Dox and 5 ng/ml Act D. This was associated with increased expression of GFP as well as considerable accumulation of p53 and its target p21 (Figure 1D). Of note, 5 ng/ml Act D exhibited the highest induction of lytic reactivation when combined with Dox; yet, it resulted in a relatively low accumulation of p53. In accord with previous reports of redistribution of nucleolar proteins during nucleolar stress induced by Act D treatment, translocation of the nucleolar transcription factor UBF into cap-like structures was evident following Act D treatment (Supplementary Figure 1). Taken together, these findings suggest that nucleolar stress, induced by Act D, synergizes with K-Rta to reactivate KSHV from latency in iSLK cells.

### Nucleolar stress produced by CX-5461 or BMH-21 enhances lytic reactivation of KSHV

**Figure 2:**
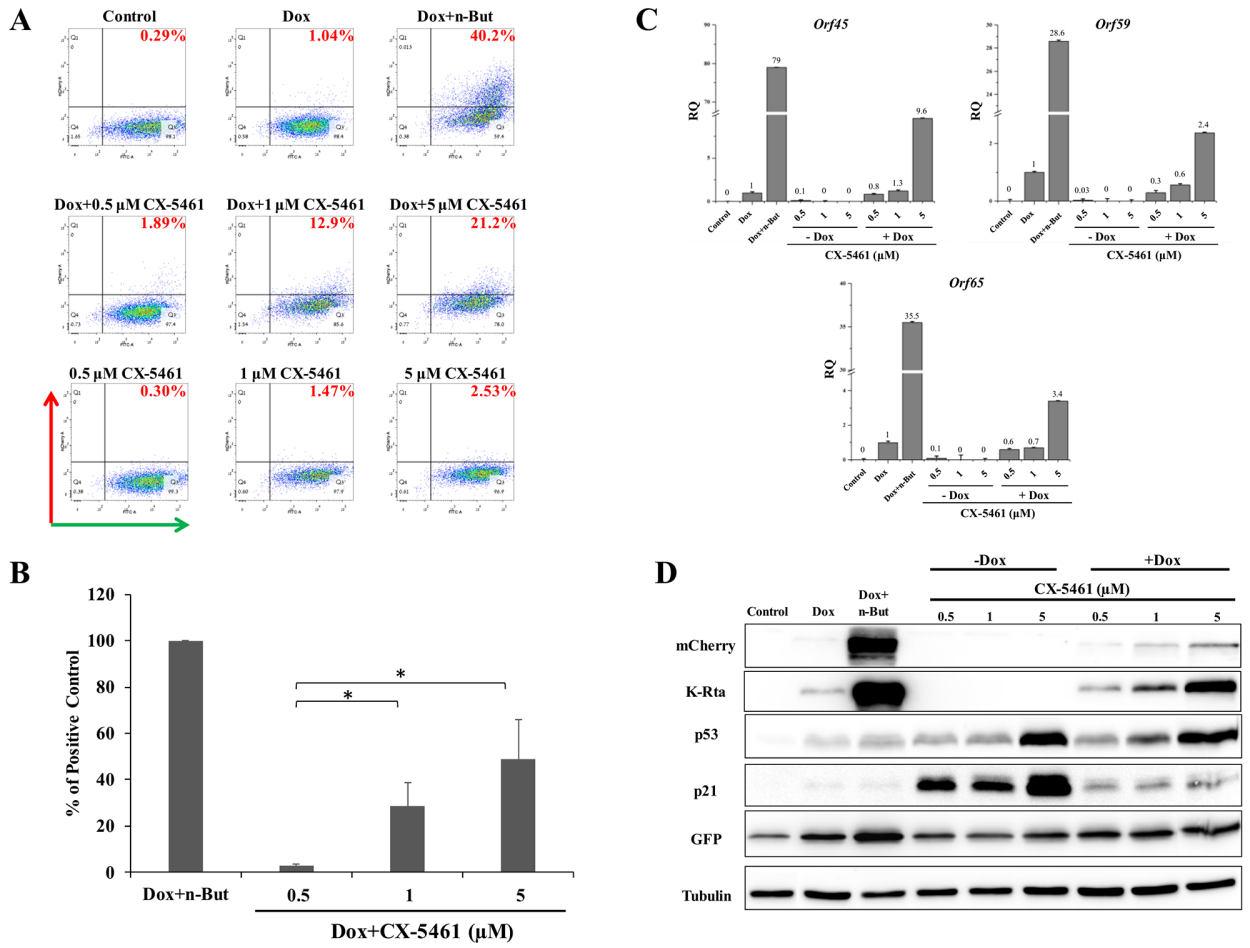
Enhancement of lytic induction of KSHV by combined treatment with CX-5461 and doxycycline in iSLK cells BAC16-mCherry-ORF45-infected iSLK cells were left untreated or treated with increasing doses of CX-5461 (0.5, 1, 5 μM) alone or in combination with 1μg/ml doxycycline (Dox) for 48 hours. Results are presented as detailed in Figure 1 (except for western blot analysis with antibody to K-Rta instead of K8).

**Figure 3:**
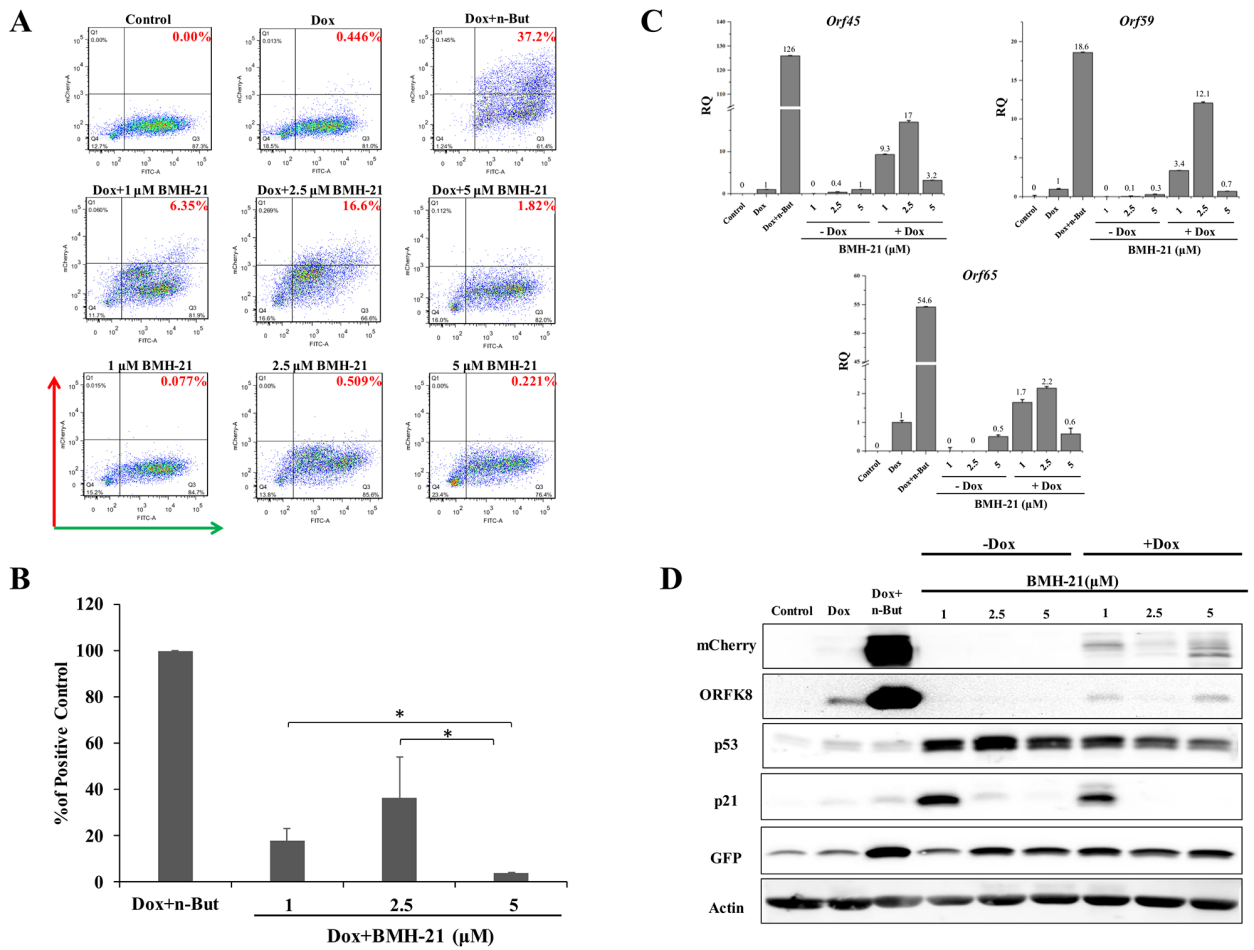
Enhancement of lytic induction of KSHV by combined treatment with BMH-21 and doxycycline in iSLK cells BAC16-mCherry-ORF45-infected iSLK cells were left untreated or treated with BMH-21 (0.5, 2.5, 5 μM) alone or in combination with 1μg/ml doxycycline (Dox) for 48 hours. Results are presented as detailed in Figure 1.

In view of the effect of Act D on lytic reactivation of KSHV, we wished to explore the effect of additional inducers of nucleolar stress on KSHV reactivation. We selected two compounds, CX-5461 and BMH-21. CX-5461 is a potent selective inhibitor of rRNA synthesis. It is a small molecule that blocks SL-1 from binding to rDNA, thereby preventing initiation of rRNA synthesis by RNA pol I [52, 53]. BMH-21 is also a small molecule, which binds GC-rich sequences to selectively inhibit RNA polymerase I, and also causes degradation of the RNA polymerase I catalytic subunit, RPA194 [54, 55]. We initially examined the effects of different doses of these compounds aiming to achieve lytic reactivation but avoiding cell death or toxicity. The concentrations shown here represent optimal doses, which produced the highest levels of lytic reactivation. We treated KSHV-infected iSLK cells with 0.5, 1, and 5 μM of CX-5461 or 1, 2.5, and 5 μM of BMH-21 alone or in combination with Dox, and assayed virus reactivation as described above by FACS analysis of mCherry expressing cells, RT-qPCR for viral lytic transcripts, and western blot analysis (Figures 2 and 3). In line with the results obtained with Act D, treatment with CX-5461 or BMH-21 alone at different concentrations failed to induce lytic reactivation of KSHV (Figures 2 and 3). However, CX-5461 and BMH-21 synergized with Dox and enhanced lytic reactivation with the highest activity at 5 μM and 2.5 μM, respectively. Interestingly, treatment with 5 μM CX-5461 produced maximum accumulation of p53 along with increased expression of its target p21, yet addition of Dox largely decreased the level of p21 (Figure 2D). In accord with previous results, redistribution of UBF was evident following CX-5461 and BMH-21 treatments (Supplementary Figure 1). Taken together, our results suggest that nucleolar stress does not induce lytic virus reactivation of KSHV, yet it synergizes with K-Rta to enhance reactivation.

### Enhancement of lytic reactivation by nucleolar stress is not dependent on p53

**Figure 4:**
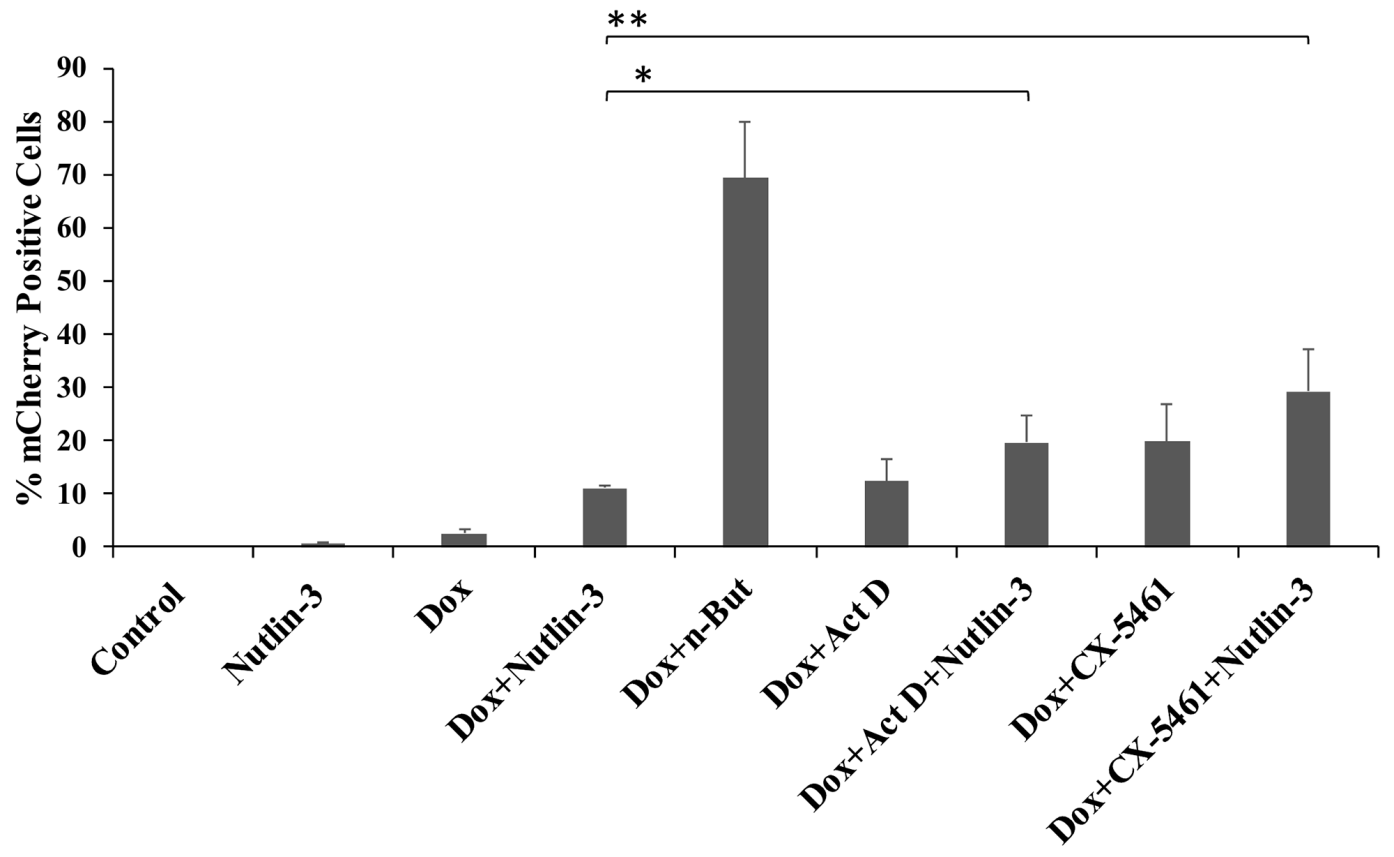
Combinations of Nutlin-3 and inducers of nucleolar stress produce additive levels of lytic reactivation BAC16-mCherry-ORF45-infected iSLK cells were left untreated (control), or treated with the indicated compounds. Treatment with 7 μM Nutlin-3 alone, 1μg/ml doxycycline (Dox) alone and combined treatment with Dox and 1 mM n-Butyrate (Dox+n-But) were used as controls. The concentrations of Act D and CX-5461 were 5 ng/ml and 5 μM, respectively. Lytic reactivation was assayed after 48 hours by FACS analysis of mCherry expressing cells. Results show the percentages of mCherry-positive cells, and represent the average of three independent experiments ±s.d. The combined treatments of Dox/Nutlin-3 + 5 ng/ml Act D, and Dox/Nutlin-3 + 5 μM CX-5461 resulted in significantly higher percentages of mCherry-positive cells as compared with Dox/Nutlin (^*^p=0.04, ^**^p=0.009).

Our initial hypothesis was that nucleolar stress, which is known to reduce the activity of MDM2 and to promote accumulation of p53, would induce lytic reactivation. To determine whether p53 is crucial for the synergistic effect of nucleolar stress with K-Rta towards lytic reactivation, we employed three approaches. First, we examined in iSLK cells the combined effect of p53 activation by Nutlin-3 treatment together with nucleolar stress. As shown in Figure 4, FACS analysis of mCherry-positive cells revealed that treatment with Nutlin-3 alone induced a minor increase in lytic reactivation, which was enhanced when combined with Dox treatment. This result is in agreement with a previous report [45]. Combined treatment with Nutlin-3 and Dox together with induction of nucleolar stress with Act D or CX-5461 resulted in a further additive increase in the fraction of cells expressing mCherry. This indicates that nucleolar stress and Nutlin-3 enhancement of lytic reactivation act through independent pathways, and therefore suggests that nucleolar stress supports lytic reactivation via a p53-independent mechanism.

**Figure 5:**
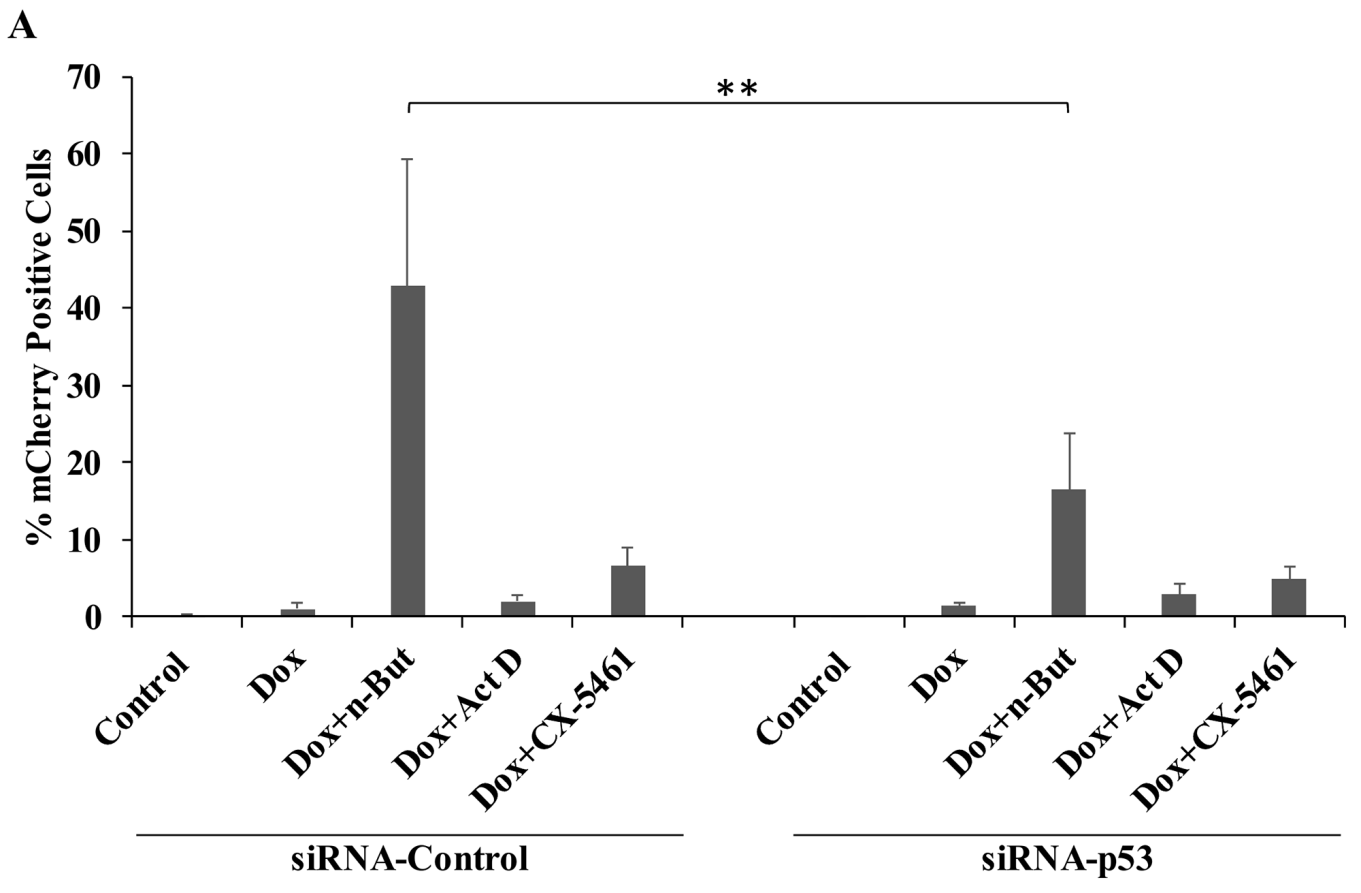

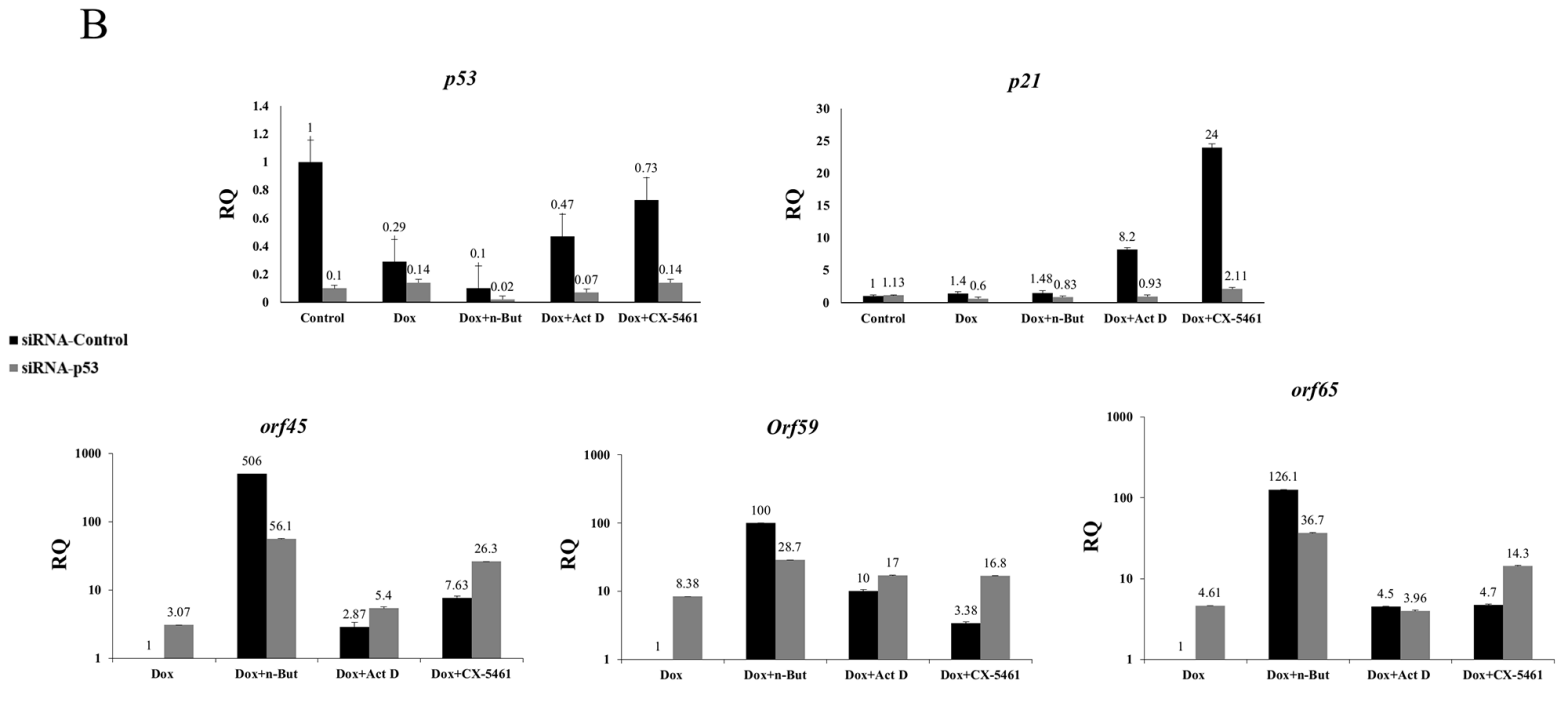
Knock-down of p53 does not impair the enhancement of lytic reactivation by nucleolar stress **(A)** BAC16-mCherry-ORF45-infected iSLK cells were transfected with control siRNA (siRNA-Control) or with siRNA which targets p53 (siRNA-p53). Cells were treated 24 hours after transfection with the indicated compounds (1μg/ml doxycycline (Dox), 1 mM n-Butyrate, 5 ng/ml Act D, 5 μM CX-5461), and lytic reactivation was assayed after 48 hours by FACS analysis of mCherry-positive cells (Results show the percentages of mCherry-positive cells and represent the average of three independent experiments ±s.d.). In cells incubated with Dox+n-Butyrate the percentage of mCherry-positive cells was significantly higher in cells that were transfected with control siRNA, as compared with cells that were transfected with siRNA targeting p53 (p=0.005). No significant difference was found between siRNA control and siRNA p53 transfected cells that were treated with Dox alone, Dox and 5 ng/ml ActD, or Dox and 5 μM CX-5461. **(B)** RT-qPCR for p53 and its target p21 confirming knock-down of p53 and of p21 induction upon nucleolar stress in cells that were transfected with control siRNA. Quantification of the viral lytic transcripts *orf45*, *orf59* and *orf65* was used to evaluate the levels of lytic reactivation. p53 and p21 mRNA abundance is presented relative to control untreated cells, while abundance of the lytic genes is shown relative to Dox treatment, which was set to 1 after normalization.

As a second approach, we knocked down p53 using siRNA and examined lytic reactivation by FACS analysis of mCherry-positive cells and RT-qPCR for viral lytic genes. As shown in Figure 5, knock-down of p53 in iSLK cells significantly reduced lytic reactivation that was induced by treatment with Dox+n-Butyrate. Yet, knock down of p53 did not have a significant effect on the enhancement of lytic reactivation by Act D or CX-5461 treatments. These findings indicate that unlike the induction of lytic virus reactivation by Dox+n-Butyrate, the enhancement of lytic reactivation by nucleolar stress does not involve p53.

Finally, we employed HCT-116 cells expressing wild-type p53 (p53+/+), and an isogenic line lacking full-length p53 expression (p53-/-). Of note, although HCT-116 cells are not natural targets of KSHV infection, they could efficiently establish latent infection and undergo efficient lytic reactivation that was associated with virion production. HCT-116 cells were infected with the recombinant BAC16-mCherry-ORF45, and lytic reactivation was initiated using a recombinant baculovirus expressing K-Rta (BacK50) under the control of an immediate-early lytic CMV promoter. Concomitantly, the cells were treated with Act D, CX-5461 or BMH-21 at different concentrations, and assayed after 48 hours for virus reactivation, as described above. As shown in Supplementary Figures 2 - 4, treatments with Act D, CX-5461 and BMH-21enhanced the effect of K-Rta expression, and higher percentages of mCherry-positive cells were detected 48 hours after treatment in both p53+/+ or p53-/- cells. These findings were supported by RT-qPCR for viral lytic transcripts. IFA confirmed redistribution of UBF following Act D, CX-5461 and BMH-21 treatments (Supplementary Figure 5). These results indicate that induction of nucleolar stress enhances lytic activation of KSHV through a pathway not involving p53.

## DISCUSSION

The nucleolus is a subnuclear compartment whose primary function is the biogenesis of ribosomal subunits. The integrity of ribosomal subunit biogenesis is essential for maintaining sufficient protein translation, and serves as an important checkpoint for cell homeostasis. Various external and internal stress conditions, such as hypoxia, nutrient deprivation or metabolic perturbations, can interfere with normal ribosomal biogenesis and trigger nucleolar stress which is often coupled with redistribution of nucleolar proteins and changes in nucleolar morphology. The primary mechanism involved in nucleolar stress responses is the formation of interactions between redistributed nucleolar proteins and the major negative regulator of p53, MDM2, which consequently leads to p53 stabilization and accumulation [50, 56, 57]. An alternative proposed mechanism is that nucleolar stress results in increased translation of mRNA encoding ribosomal proteins containing a 5’-terminal oligopyrimidine tract (5’-TOP), such as RPL11, thereby generating an excess of unassembled ribosomal proteins that are free to bind MDM2 and to inhibit its activity [58]. Yet, p53-independent response pathways, involving other regulators such as E2F-1, p27kip1, and PIM1, can be initiated by nucleolar stress and alter cell fate [50, 59]. Nucleolar stress offers a target for cancer therapy, and compounds that specifically target this pathway, such as CX-5461 and BMH-21, are being considered as promising drug candidates [60–62]. In fact, several anti-cancer drugs that are widely used, including cytotoxic chemotherapy, interfere with ribosomal DNA (rDNA) transcription or ribosomal RNA (rRNA) processing, but to date, the contribution of these effects beyond the general inhibition of cancer cell growth remains to be determined [56, 63, 64].

The tumor suppressor p53 is a major regulator of cell cycle progression and cell death. Activation of the p53 pathway by using Nutlin-3, a small molecule inhibitor which targets the interaction of p53 with its negative regulator MDM2, was previously shown to induce cell cycle arrest and massive apoptosis in KSHV-infected PEL, U2OS, SLK and endothelial cells [47, 65]. Mechanistically, this treatment was shown to disrupt the inhibitory complex between p53 and the KSHV latent protein, LANA-1, leading to restoration and activation of the p53 pathway [65]. Concomitantly, Nutlin-3 treatment was shown to reactivate the lytic cycle of KSHV. In addition, an siRNA screen for regulators of KSHV reactivation identified MDM2 as a negative regulator of KSHV reactivation, whose depletion promotes reactivation [66]. Interestingly, this study identified p53 as a regulator of virus reactivation, yet its effect was found to be mediated through the activation of cellular rather than viral genes. Additional experiments have shown that efficient reactivation of KSHV requires induction of p21, through p53-dependent and independent mechanisms [66]. Further studies by Chang et al. [45] revealed MDM2 as a negative regulator of KSHV reactivation, which directly interacts with K-Rta to target its degradation; accordingly, MDM2 knock-down enhances expression of K-Rta and lytic activation. Of note, although p53 mutations are frequent in human cancers, they are rarely found in KSHV-associated malignancies [66–68].

In the present study, we interfered with ribosomal RNA transcription to induce nucleolar stress by treatment with Actinomycin D, CX-5461 or BMH-21. In accordance with the reported activities of these drugs, alterations in nucleolar morphology, coupled with increased levels of p53, were documented. These responses were generally dose-dependent. Treatment with these compounds alone, at different concentrations, did not stimulate lytic virus reactivation. However, a dose dependent enhancement of lytic cycle induction by these compounds was evident when combined with expression of the K-Rta protein. Further experiments employing treatments with Nutlin-3, knock-down of p53 and isogenic p53+/+ and p53-/- cells indicated that in contrast to Nutlin-3, which induces lytic reactivation through p53 restoration, induction of lytic reactivation by nucleolar stress does not depend on p53. Thus, nucleolar stress does not initiate K-Rta expression, but can enhance lytic virus reactivation in combination with additional inducer/s that generate initial K-Rta expression, which is subsequently enhanced.

It was proposed that an “emergency” lytic activation pathway of KSHV can be induced through an alternative K-Rta-independent program that is triggered by caspase-3 activation and provides the virus a chance to reproduce before completion of host cell apoptosis [69–71]. However, our results suggest that the effect of nucleolar stress on lytic reactivation requires the expression of K-Rta. The effectiveness of nucleolar stress as lytic inducer was far below that of n-Butyrate, which is known as a very potent inducer of KSHV reactivation in most experimental KSHV models. Of note, we harvested cell media from iSLK cells 48, 72 and 96 hours following treatments and examined virus production by reinfecting naïve cells and enumerating cells expressing GFP. As expected, increasing quantities of infectious virions were detected in media that were collected from cells that were treated with increasing concentrations of Dox alone (0.25, 0.5, 1, 2.5 and 5 μg/ml) while combined treatments with Dox and n-Butyrate yielded far higher quantities of infectious particles. Yet, we failed to detect infectious progeny in supernatants collected from cells subjected to the other treatments comprising of Dox together with inducers of nucleolar stress (data not shown). This could be due to limited enhancement of lytic reactivation, but most likely reflects an incomplete lytic phase. Nevertheless, under physiological conditions several layers of signaling operate simultaneously to control virus latent/lytic infection and enhancement of lytic induction by nucleolar stress could tilt the balance towards full lytic induction.

Taken together, our study identifies nucleolar stress as a novel regulator of KSHV infection, which synergizes with K-Rta to enhance lytic reactivation. Act D, CX-5461 and BMH-21 share the potential to induce nucleolar stress, yet they differ in their biochemical activities. Therefore, the mechanism by which these compounds enhance lytic reactivation of KSHV could also differ. Our study suggests that certain therapeutic interventions, that induce nucleolar stress may increase circulating KSHV and thus can have considerable clinical consequences on the outcome of KSHV infection. Furthermore, KSHV-related neoplasms often require cytotoxic chemotherapy with drugs that can potentially cause nucleolar stress; our results indicate that these drugs may induce lytic replication of KSHV.

## MATERIALS AND METHODS

### Cells, viruses and siRNA

SLK and iSLK-PURO cells (kindly provided by Prof. Don Ganem, (University of California) and Prof. Rolf Renne (University of Florida)) [46] were maintained in Dulbecco’s modified Eagle’s medium (DMEM) supplemented with 10% fetal calf serum (FCS), 50 IU/ml penicillin and 50 μg/ml streptomycin (Biological Industries, Kibbutz Beit Haemek, Israel). To maintain the Tet-On transactivator and K-Rta expression cassette, 250μg/mL G418 and 1μg/ml puromycin (A.G. Scientific Incorporation), respectively, were added to iSLK-PURO growth medium [46]. HCT-116 colorectal carcinoma cells expressing wild-type p53 (p53+/+) or lacking the two p53 alleles (p53-/-) (kindly supplied by Prof. Moshe Oren, Weizmann Institute, Israel) were grown McCoy’s 5A medium supplemented with 10% FCS [72]. BAC16-mCherry-ORF45 recombinant virus has been previously described [48]. Infected iSLK-PURO and HCT-116 cells were selected with 600 and 500 μg/ml Hygromycin (Sigma), respectively. Baculovirus encoding K-Rta (BacK50) (kindly provided by Prof. David Lukac, Rutgers University, NJ) was propagated in Sf9 cells, as described previously [73] and titrated to obtain efficient reactivation in combination with n-Butyrate.

To measure the effect of nucleolar stress, 5x10^5^cells were plated on 6-well plates. After 24 hours, cells were left untreated or treated with 1 μg/ml Doxycycline (Dox) (Sigma), or 1mM n-Butyrate (Sigma), Actinomycin D (Sigma), CX-5461 (AdooQ BioScience), BMH-21 (Sigma), or Nutlin-3 (Cayman Chemical Company, MI, USA), and incubated for 48 hours. The nonspecific siRNA control (Stealth siRNA negative control hi GC 12935-400) and siRNA against p53 (TP53VHS40366 Cat No. 10620318 and 10620319) were from Invitrogen. Cells were transfected using Lipofectamine RNAiMAX (Invitrogen), and reactivated 24 hours after transfection by the indicated treatments for 48 hours.

### FACS quantification of cells undergoing lytic induction

Cells were trypsinized, washed and suspended in 0.5 ml PBS; 0.5 ml 2% formaldehyde was added, and the cells were incubated at 4°C for 20 min. Cells were then washed with PBS, and GFP and mCherry-positive cells were determined by fluorescence-activated cell sorting (FACS) (BD FACSAria III, BD Biosciences). Dead cells were excluded based on side and forward scatter profiles. Data analysis was performed using FlowJo software.

### Real-time reverse-transcription (RT)-PCR

Total RNA was extracted by EZ-RNA Total RNA Isolation kit (Biological Industries, Israel). Residual DNA contamination was eliminated by DNase treatment (Turbo DNA-free kit (Ambion)). cDNA was generated from a total of 1 μg RNA with the qScript cDNA Synthesis kit (Quanta Biosciences) and priming with random hexamers according to the manufacturer’s instructions. Real-time quantitative polymerase chain reaction (RT-qPCR) was performed in a total volume of 10 μl with 1.5 μl of 1/2-diluted cDNA, 0.15 μM of each primer specific for viral mRNA, and Fast Syber Green Master mix (Applied Biosystems). The expression levels obtained for each gene were normalized to those of beta-actin. The primers used were previously described [74]. All PCR reactions were run in triplicates on StepOne Plus Real-Time PCR System (Applied Biosystems Inc., Carlsbad, CA).

### Antibodies and western blot analysis

Cells were washed twice in cold PBS, suspended in RIPA lysis buffer, and incubated on ice for 30 min. Cell debris were removed by centrifugation at 12,000g for 15 min at 4°C. Protein lysates were resolved by SDS-PAGE and transferred to nitrocellulose membranes using Trans blot turbo RTA midi nitrocellulose transfer kit (BioRad). The protein content of different samples was verified by Ponceau S staining. The nitrocellulose membranes were blocked with 5% dry milk in TBS, and subsequently incubated with primary mouse antibodies to GFP (Covance Research Products), mCherry (catalog No. 632496, Clontech), ORF K8 [75] (a kind gift from Prof. Yan Yuan, University of Pennsylvania), K-Rta [76] (kindly provided by Prof. Keiji Ueda, Osaka University, Japan), GAPDH (sc-25778, Santa Cruz), Tubulin (E7-S, DSHB), p53 (sc-126, Santa Cruz) and p21 (sc-6246, Santa Cruz). Reactive bands were detected using anti-mouse conjugated to horseradish peroxidase, and visualized using the EZ-enhanced chemiluminescence (ECL) detection kit (Clarity Western ECL Substrate, BioRad).

### Fluorescence microscopy

Cells were seeded on coverslips, washed with PBS, and fixed by incubation in 4% formaldehyde for 20 min at room temperature. The cells were then washed twice in PBS and permeabilized in PBS containing 0.2% Triton x100 and 1% BSA at room temperature for 30 min. Cells were probed with primary antibody to UBF (sc-13125, Santa Cruz), and conjugated secondary anti-mouse Alexa Flour 647 (cat No. 705-605-147, Jackson Labs) antibodies were then applied for detection. To stain the nuclei, the cells were incubated for 30 min with 0.05 μg/ml Hoechst dye (Sigma). Cells were examined and photographed under a confocal laser-scanning microscope (Zeiss LSM 510 META, or Leica STED Live Imaging).

## SUPPLEMENTARY MATERIALS FIGURES



## Abbreviations

KSHV: Kaposi’s sarcoma-associated herpesvirus
K-Rta: KSHV replication and transcription activator
Actinomycin D: Act D
Doxycycline: Dox
