# Supplementary material for: Nucleolar stress enhances lytic reactivation of the Kaposi’s sarcoma-associated herpesvirus
Source: Oncotarget. 2018 Feb 15;9(17):13822–33. doi: 10.18632/oncotarget.24497 (PMC5862618; doi:10.18632/oncotarget.24497)
Supplement: Supplementary file 1 [file oncotarget-09-13822-s001.pdf]

Nucleolar stress enhances lytic reactivation of the Kaposi's sarcoma-associated herpesvirus

SUPPLEMENTARY MATERIALS

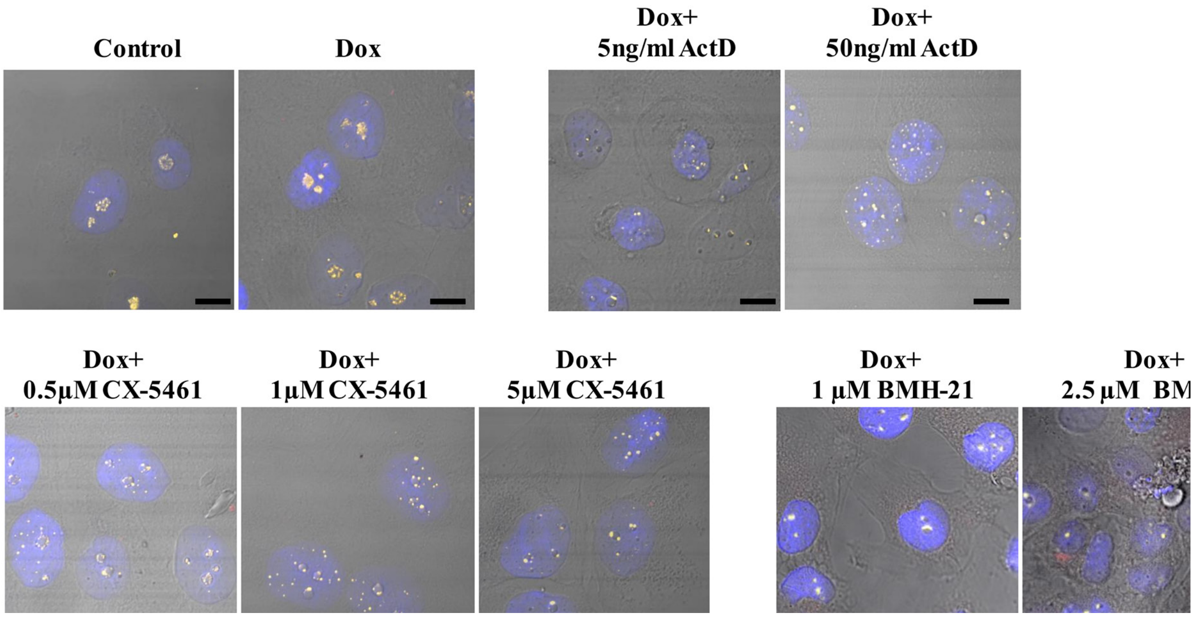

Supplementary Figure 1: Immunofluorescence showing nucleolar disruption following treatment with Actinomycin D, CX-5461 and BMH-21. BAC16-mCherry-ORF45-iSLK were treated with the indicated compounds. Nucleolar disruption was verified by immunofluorescence staining of the nucleolar protein, UBF. Scale bar 10 µm.

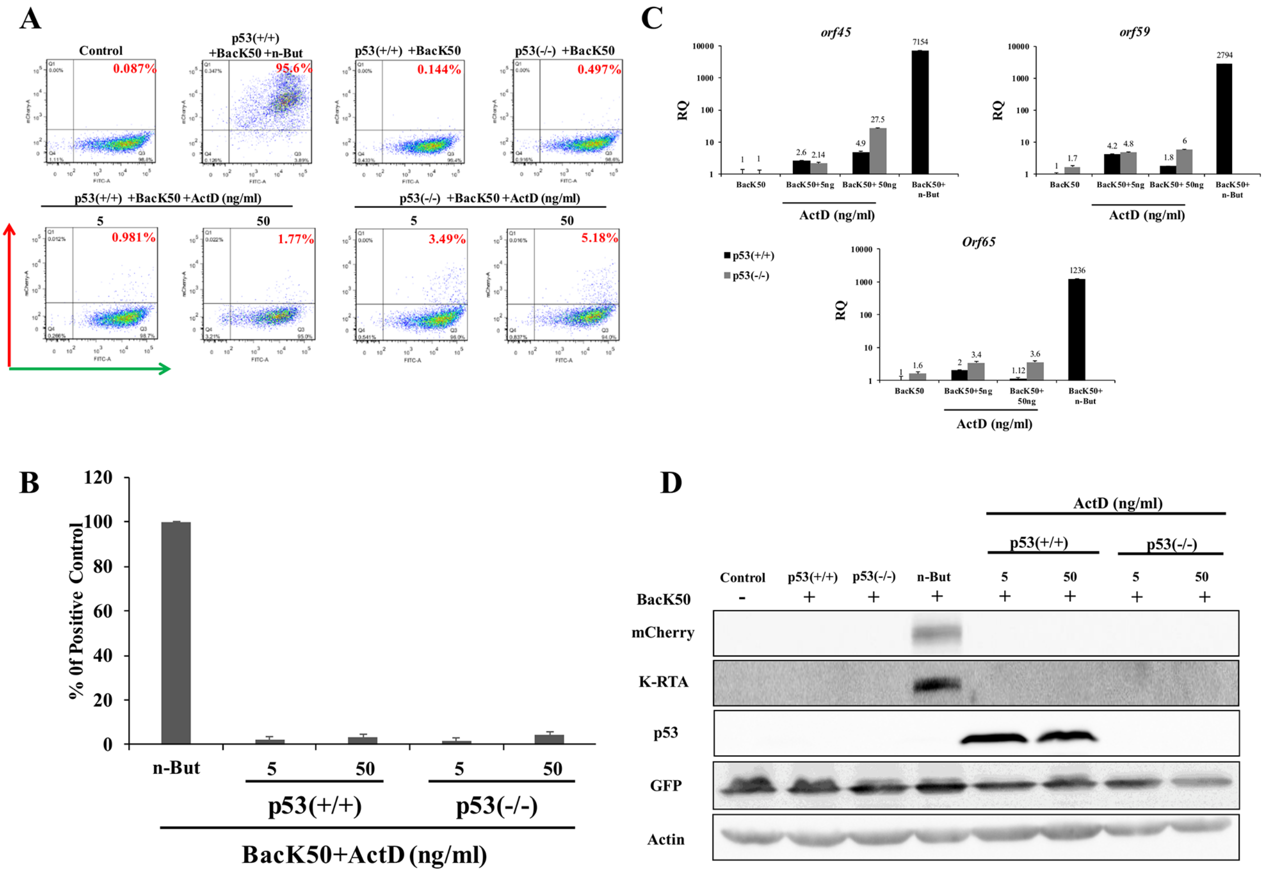

Supplementary Figure 2: Treatment with Actinomycin D combined with recombinant K-Rta-baculovirus (Back50) transduction enhances lytic reactivation of KSHV in HCT-p53+/+ and p53-/- cells. BAC16-mCherry-ORF45-p53+/+ and p53-/- infected HCT-116 cells were left untreated (control) or treated with Act D (5 or 50 ng/ml) in combination with Back50 transduction. Lytic virus reactivation was assayed as described in Figure 1 after 48 hours.

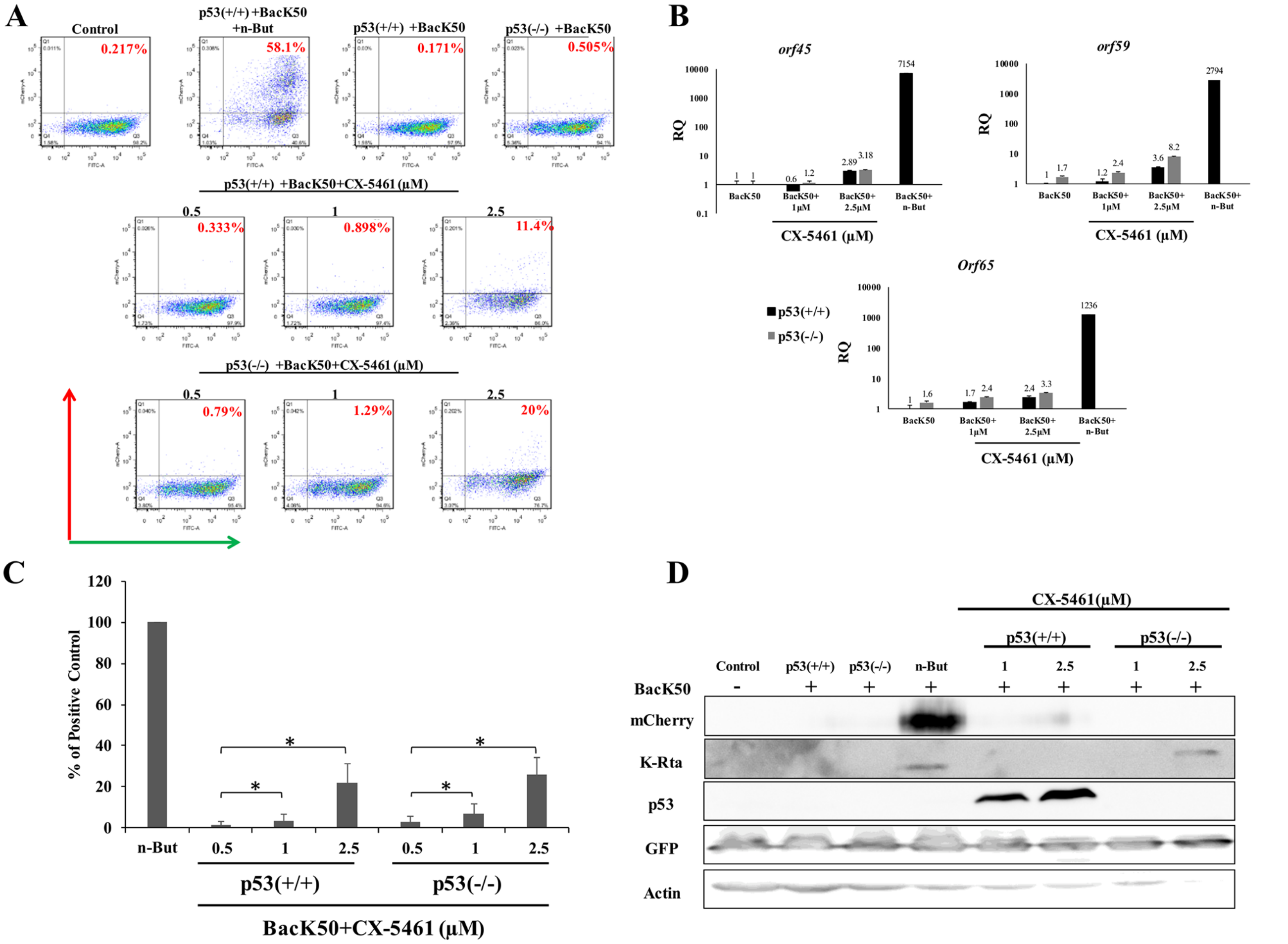

Supplementary Figure 3: Treatment with CX-5461 combined with recombinant K-Rta-baculovirus (Back50) transduction enhances lytic reactivation of KSHV in HCT-p53+/+ and p53-/- cells. BAC16-mCherry-ORF45-p53+/+ and p53-/- infected HCT-116 cells were left untreated (control) or treated with CX-5461 (1 or 2.5 µM) in combination with Back50 transduction. Lytic virus reactivation was assayed as described in Figure 1 after 48 hours.

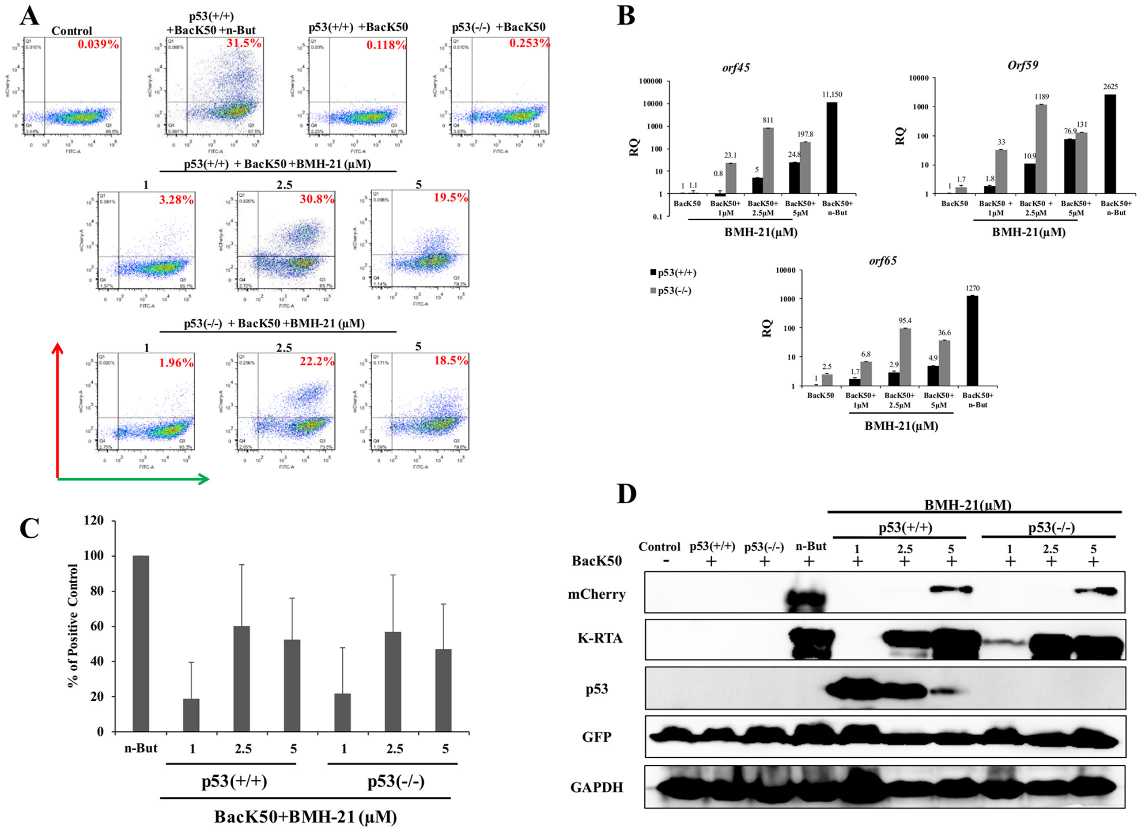

Supplementary Figure 4: Treatment with BMH-21 combined with recombinant K-Rta-baculovirus (Back50) transduction enhances lytic reactivation of KSHV in HCT-p53+/+ and p53-/- cells. BAC16-mCherry-ORF45-p53+/+ and p53-/- infected HCT-116 cells were left untreated (control) or treated with BMH-21 (1, 2.5 and 5 µM) in combination with Back50 transduction. Lytic virus reactivation was assayed as described in Figure 1 after 48 hours.

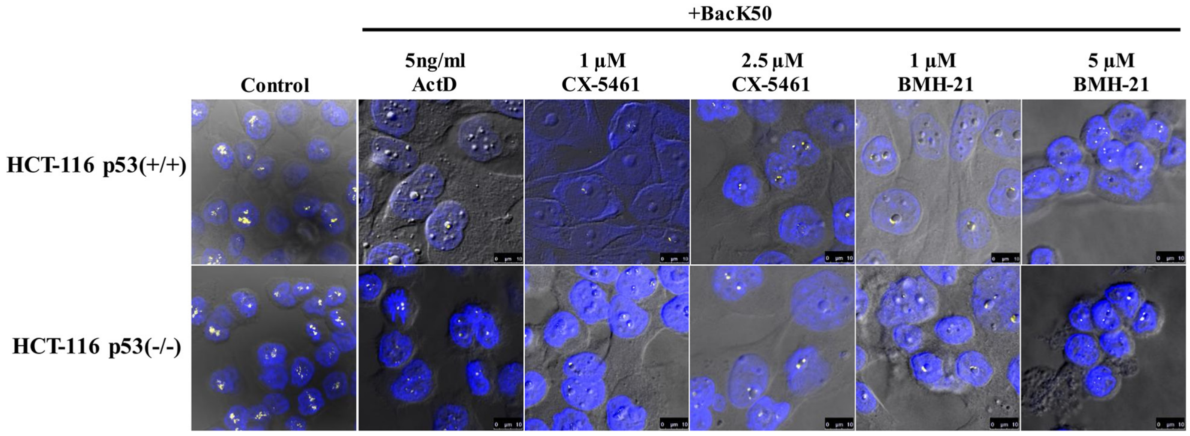

Supplementary Figure 5: Immunofluorescence showing nucleolar disruption following treatment with Actinomycin D, CX-5461 and BMH-21. p53+/+ and p53-/- HCT-116 cells were treated with the indicated compounds. Nucleolar disruption was verified by immunofluorescence staining of the nucleolar protein UBF. Scale bar 10 µm.
